# Changes in the determinants and spatial distribution of under-five stunting in Bangladesh: Evidence from Bangladesh Demographic Health Surveys (BDHS) 1996–97, 2014 and 2017/18

**DOI:** 10.1371/journal.pone.0278094

**Published:** 2022-12-01

**Authors:** U. R. Saha, C. F. A. van Wesenbeeck

**Affiliations:** 1 Department of Public Health, Erasmus MC, University Medical Center Rotterdam, Rotterdam, The Netherlands; 2 Amsterdam Centre for World Food Studies, VU University Amsterdam, Amsterdam, The Netherlands; VART Consulting PVT LTD, INDIA

## Abstract

**Background:**

Bangladesh has experienced tremendous change in child nutrition over the past few decades, but there are large differences between different regions in progress made. The question is whether continuation of current policies will bring the progress needed to reach national and international targets on child nutrition security.

**Data and methods:**

Using national data BDHS 1996/97, 2014, and 2017, this study attempts to map such reductions across Bangladesh and to explore the distribution of covariate effects (joint effects) that are associated with childhood stunting over these two periods, overall and by region. The main contribution of this paper is to link observed stunting scores to a household *profile*. This implies that different variables are evaluated jointly with stunting to assess the likelihood of being associated with stunting.

**Results:**

Overall, the covariates: *‘Parental levels of education’*, *‘children older than one year old’*, *‘children live in rural area’*, *‘children born at home’* formed the country winning profile in 1996/97, whereas parental levels of education disappear in the winning profile for children stunted in 2014. This implies that over the years, Bangladesh has been successful in addressing parental education for long-term reductions in child undernutrition. In addition, the diversity of profiles of households with stunted children increases over time, pointing at successful targeting of policies to increase food security among children over the period. However, in areas where improvements have been insignificant, also the profiles remain stable, indicating a failure of policies to reach the target populations. The analysis for 2017 confirms this picture: the diversity of profiles remains high, with little change in the dominant profiles.

**Conclusion:**

Further decline in stunting is possible through region specific multipronged interventions, targeting children older than one year among vulnerable groups, in addition with strengthening family planning programs as larger families also have a higher risk to have stunted children. In general, the profiles in 2014 and 2017/18 are much more diverse than in 1996, which can be explained by the relative success of specific targeted policies in some divisions, while being much less successful in other regions. In sum, our results suggest that the challenge lies in the *implementation* of policies, rather than in the generic approach and assumed theory of change.

## 1. Introduction

Globally, nutritional status measured as length-for-age/height-for-age (stunting) is one of the predictors of the well-being of young children. The World Health Organization has identified malnutrition as the underlying cause of close to half of child deaths worldwide [1, 2]. In developing countries annually about 13 million under-fives die of causes linked to malnutrition [3]. Stunting affects children not only by increasing deaths among them and making them more susceptible to disease; it also negatively influences children’s behavior. There is strong evidence that children who are suffering from linear growth failure are more likely to be affected by infectious disease, for example, malaria, diarrhea, and pneumonia [4], and poor nutrition during childhood causes irreversible damage to cognitive development and future health. Strong associations are observed between child malnutrition and less schooling and reduced economic productivity [5–7] estimate that 2.91 years of performance deficit that resulted from stunting caused upto 19.8% loss of adult income. It also appears that damage grieved in early life can lead to permanent impairment and it may also affect future generations. For example, malnourished girls often grow up to become malnourished mothers themselves with detrimental impacts such as low birth weight on their offspring.

In the recent past, Bangladesh has made notable achievements in the development indicators of child health. Under-five mortality has declined in Bangladesh from 133 per thousand in the mid-90s to 46 in recent years [8, 9]. The rate of stunting (length-for-age/ height- for- age<-2 SD) among under-five children—an indicator of the state of the chronic undernutrition—in Bangladesh has come down from 55% (59% WHO new) in 1996–97 to 29% (36% WHO new) in 2014 and 23% (31% WHO new) in 2017/18. However, despite of such reductions, still almost one-quarter of all under-fives suffer from stunting, accounting for over 5 million children. Earlier studies on stunting in Bangladesh rarely focus on both determinants and spatial variation of stunting as indicator of childhood malnutrition. Studies frequently report on the effects of socioeconomic and demographic factors that are likely to be associated with stunting prevalence in children in Bangladesh [10–16]. Heady et al., 2015 [17] performed multivariate analysis using successive DHS data sets (1997–2011). The major factors correlated with the decline in stunting in Bangladesh include a rise in household assets; improvements in parental education; a reduction in open defecation; prenatal and birth delivery care; birth order and birth intervals; and maternal height. However, these conclusions are drawn at national level and do not account for the substantial spatial variation in stunting levels and causes.

Bangladesh has made significant progress in women’s education, infrastructure and economic development, but stunting remains a serious challenge, particularly since not all areas have witnessed significant reductions in stunting rates. Hence, to improve the nutritional status of all children across Bangladesh, it is vital to understand the spatial variation of stunting as well as the local dynamics. Hence, this paper studies the spatial variation of stunting, as well as spatial patterns of explanatory factors, in the periods 1996–97–2014. In addition, we analyzed BDHS 2017/18 data to compare the dynamics of the changes in the exploratory factors between periods 1997–2014 and 2014–2017.

The large literature on determinants of stunting report that age and gender of the child, birth weight and birth interval; mother’s education and nutritional status; household economic status and family size; and residential place are significant predictors of stunting. The paper considers the risk factors for stunting in a spatially explicit analysis and compare our findings for 1996/97 with those for 2014 and 2017. These highlights possible shifts in critical factors associated with stunting over time. The findings may help to meet the goals of reducing stunting at national and international targets that Bangladesh has committed to.

Bangladesh played a critical role in the second International Conference on Nutrition in 1992 and made a commitment to include nutrition policy in all development activities. As committed, the first National Food-Security and Nutrition Policy (NFNP 1997) was developed in 1997. The initial success achieved during the decade between 1997 and 2007 (the stunting rates declined by 12%, from 55% to 43%) raised the government’s ambitions, and the Health, Population and Nutrition Sector Development Programme (HPNSDP) set a target to bring down the stunting rate by approximately 25 percent by 2021 [18]. At the same time the World Health Assembly (WHA) requires Bangladesh to achieve a stunting rate of 21.6% by 2025 [19, 20], a reduction of 40%. However, the Bangladesh DHS 2011 shows that between 2007 and 2011, the stunting rate has almost stagnated (the prevalence of stunting was only 2% lower, at 41%). Stunting rates must decrease at an annual rate of 5.3 percent in the period from 2014 to 2021 if the HPNSDP target is to be achieved and at a rate of 4.8 percent in the period from 2014 to 2025 if the WHA target is to be met. By contrast, the rate at which stunting has fallen in the period from 1996–97 to 2017 is about 3 percent, much too low to meet both national and international targets. Given the past record, it is unlikely that current policies to reduce hunger and increase production will create the necessary impact. The paper’s comparison between three BDHS [8, 9, 21] may provide useful information on the impact of the National Food and Nutrition policy in 1997 and other nutrition policies after 1997 [22, 23] and add insights to the regional disparities in achieving improvements. In the discussion section, we will highlight some important messages from the findings for the formulation of future nutrition policy in Bangladesh.

Since 1998, several national programs have been launched by the Ministry of Health, and Family Welfare (MOHFW). The aim of these programs was to stimulate demand for health, population and nutrition services while also improving the quality of these services to reduce morbidity and mortality, and improve nutrition status, especially of women and children. The first Health and Population Sector Program was implemented during the period 1998–2003, followed by the second Health and Nutrition and Population Sector Program (2003–2011), the third sector-wide program HPNSDP (2011–2016), and the fourth HNPSDP (2016–2021). Policy briefs using the data from BDHS 2014 [22] highlight the importance of nutrition-specific interventions like breastfeeding, complementary feeding, micronutrient supplementation, adequate and balanced diet during pregnancy, and treatment of acute malnutrition. However, the report also revealed that the coverage of these interventions was not scaled up till the write-up of the policy briefs in 2016. Therefore, at first step we target to analyze the data sourced from BDHS 2014 covering the period between 1996–2014 before the implementation of sector-wide, holistic approach, HNPSDP program between 2016–2021. Next, we also analyze the data from the BDHS 2017/18, to address the impact of the HNPSDP program. We note that at present, the results of the 2020 BDHS are not yet available The results are discussed in the discussion section 4.

## 2. Data and methods

### 2.1. Data

**Fig 1:**
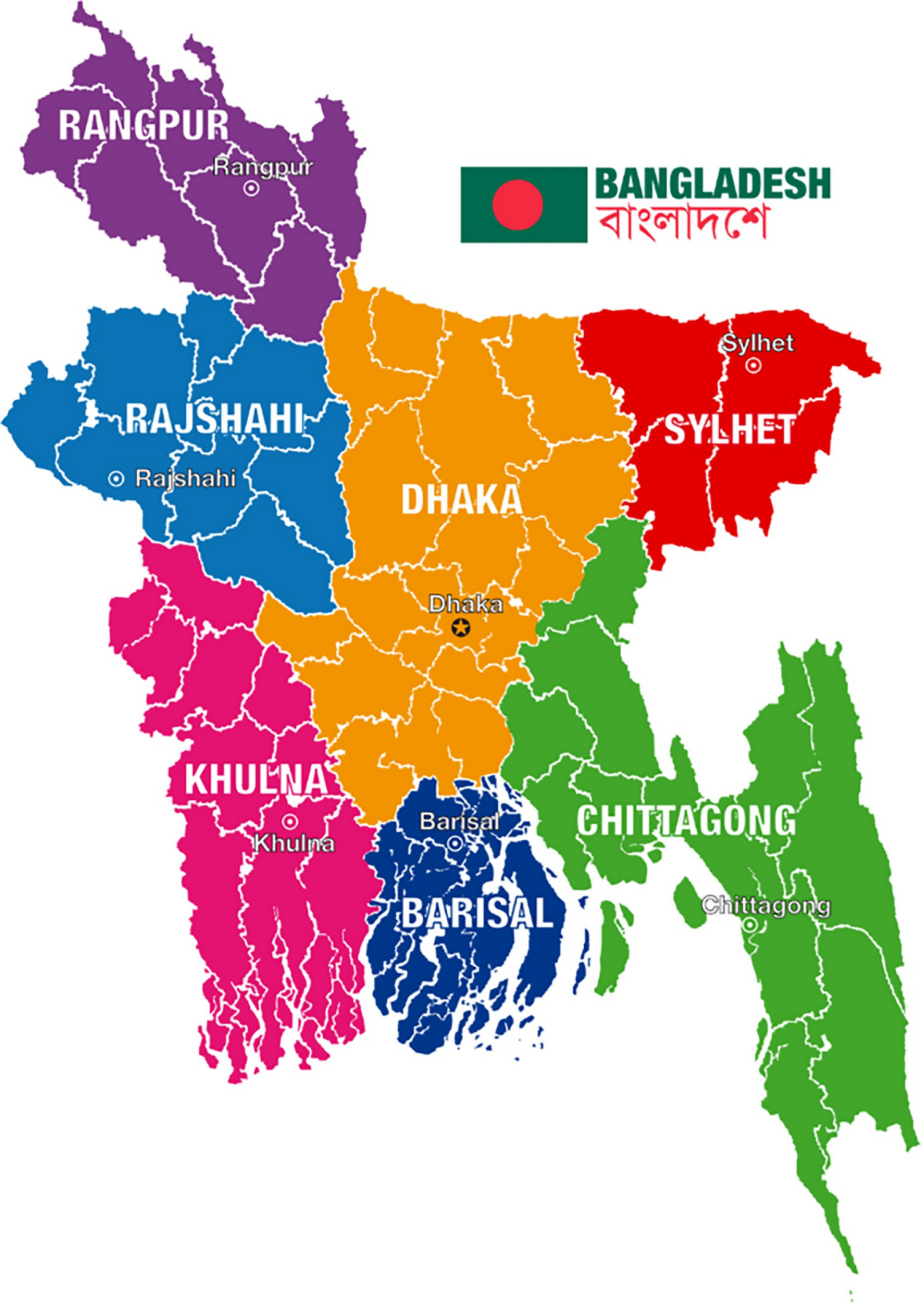
Political map of Bangladesh. Source: Bangladesh Political Map | Free SVG.

The study relies on three DHS surveys for Bangladesh (1996/1997, 2014 and 2017/18, available from DHS (2022) [24]. These datasets are national representative surveys, gathering information on a wide range of socio-demographic and health indicators of women of reproductive age (15–49), and young children aged 0–59 months. In 2017 and 2014, the households are located in 672, and 600 clusters, covering 420 Upazilas of the 492 and 335 of the 463 Upazilas with fairly large samples in each of the 8 divisions Barisal, Chittagong, Dhaka, Khulna, Mymensingh (newly formed in 2015), Rajshahi, Rangpur and Sylhet. The 1996/97 survey covered 301 clusters from 6 divisions. All surveys are cross-sectional with two-stage stratified sampling design. Census enumeration areas are selected as primary sampling units in the first stage of the sampling frame. At the second stage households are randomly selected from the primary sampling unit and ever-married women aged 15–49 years are interviewed from the selected households (for details about sampling design see [8, 9, 21]. Geographic coordinates and altitude of each cluster are recorded for the 2014 and 2017 datasets, but since the 1996 DHS is not geo-referenced, analysis is done at the lowest administrative level. For later reference, the map of Bangladesh is included in Fig 1.

Data for this study are extracted from the Stata file “Children’s Recode” of the surveys. The measurement of linear physical growth i.e. height-for-age (stunting) as indicator of chronic nutritional status is available in the data set. The dependent variable for this study is stunting (HAZ<-2 SD), i.e., being more than two standard deviations below the median (-2 SD) of the WHO reference population in terms of height-for-age. For comparison between three surveys the stunting rates for the 1996 survey are recomputed using the new WHO guidelines (the NCHS/CDC/WHO growth standard, WHO new). The study weighted sample for 1996/97, 2014 and 2017/18 surveys include 4711, 6965, and 8759 children aged 0–59 months old, respectively. The variables selected from these surveys are discussed in the Results section.

### 2.2. Characterizing households: Integrated statistical analysis

The main contribution of this section of the paper is to link observed stunting scores to a household *profile*. This implies that different variables are evaluated jointly with stunting to assess the likelihood of being associated with severe or moderate stunting. Formally, observed values of the variables used in the analysis define a joint empirical frequency distribution. Conditional frequency distributions can be derived from this joint distribution by partitioning the answers by say, *S* respondents indexed *s* into a vector *y* of dependent variables and a vector *x* of independent variables, taking the frequencies of *y* conditional on *x*. As the conditional frequencies are naturally interpreted as probability estimates, we also compute the most probable characteristics associated to each *x*-value, which can be interpreted as “winner”, in the sense of having the highest probability to have the desired *y* outcomes, as well as the runner up (second-best guess) and so on. The *coverage* of a profile is the mass of a class divided by the total mass of the relevant group, while the edge of the winning profile over the runner up is the ratio of their maximum likelihood probabilities. Formal definitions and conditions are included in Box 1 (reproduced from Van Wesenbeeck et al., 2016) [25]. Selection of the best profile is based on two criteria: (1) the *coverage* of the profile, (2) the *edge* over the runner up.

**Box 1. The polling approach**

Maximum likelihood prediction ("polling")

*c_s_* = (*c*_1*s*_,…,*c_rs_*,…,*c_Rs_*): value of *x_s_* (integer coded)

*g_s_* = (*g*_1*s*_,…,*g_rs_*,…,*g_Rs_*): value of y_*s*_ (integer coded)

*w_s_*: mass of the observation, *s* = 1,…,*S*

triple: (*y_s_,x_x_;w_s_*) defines empirical joint distribution of (*y,x*)

$n_{S_{gc}}$: number of cells in $S_{gc}^{0}\subset S$, with data on *c,g,w*

mass of a class: $m_{gc}=\frac{1}{n_{S_{gc}}}\sum_{s\in S_{gc}^{0}}w_{s}$

conditional frequency: $P_{g|c}=\frac{m_{gc}}{\sum_{g\in G_{c}}m_{gc}}$

coverage: $\frac{m_{gc}}{m_{g}}$

edge: $\frac{P_{g|c^{*}}}{P_{g|c^{**}}}$, where *P*_g|c*_ = max_*c*_ *P_g|c_*;*P*_g|c**_ = max_c≠c*_ *P_g|c_*

Given that potentially, many variables are available (say, an entire survey), restriction to a limited set of candidates is vital. Theoretical considerations can provide guidance, and univariate analysis may also be used to identify potentially important aspects that need to be included in the set used for the analysis. In addition, a balance needs to be struck between including a relatively large set of variables in the profile, with a high degree of specificity, but a low number of observations in each profile, or a small set, with a broad coverage, but less specificity.

Van Wesenbeeck et al. (2016) [25] have shown that the best results are obtained when a total of 5 variables are included in the profile. Hence, our study tests for all possible permutations of 5 variables from the total set of eligible variables identified from the literature and confirmed through univariate analysis (see section 3.2 below). Further selection of the best set of 5 variables is done by considering the coverage and edge of each of the permutations.

## 3. Results

### 3.1 Identifying and locating vulnerable groups: 1996 vs 2014

The first step is to compare the prevalence of stunting, and thereafter severe and moderate stunting throughout Bangladesh between the years 1996 and 2014 as this period was characterized by targeted interventions. To identify severe and moderate stunting, we use the WHO classification: stunting is severe if the score is below -3 standard deviations from the norm, and moderate for a score between -2 and -3 standard deviations. As mentioned earlier, stunting rates for 1996 are recomputed using the new WHO guidelines to enable comparison with the 2014 figures.

**Fig 2:**
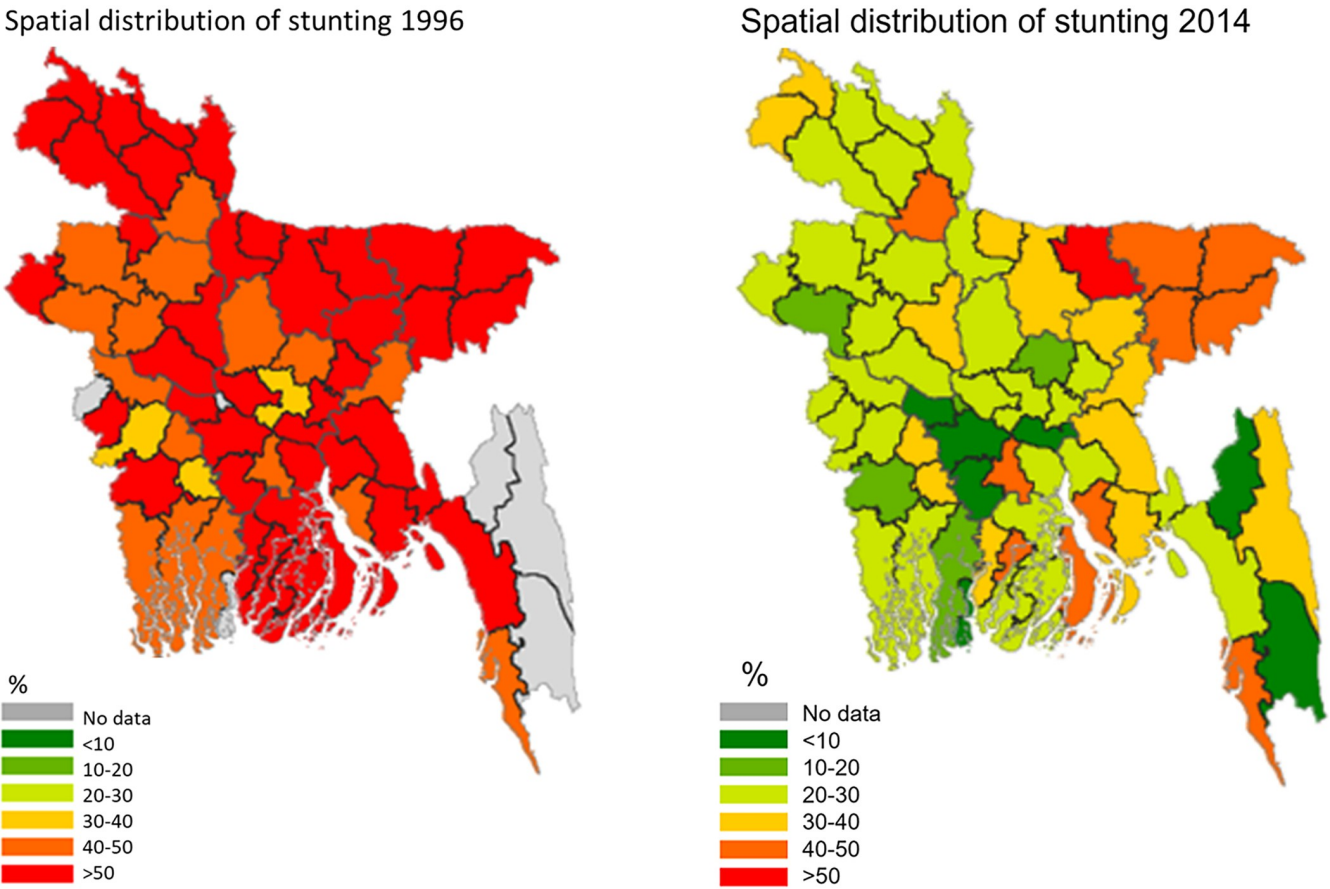
Spatial distribution of total stunting among under-fives. Source: Own calculations with GRCP software.

Fig 2 shows the district level distribution of stunting (Z<-2 SD) of under-fives for 1996 and 2014, respectively. In 1996, stunting was highly prevalent in all districts of Rangpur, Sylhet, Barisal and Mymensingh regions, and a few districts from other regions (> = 50%). However, nearly 20 years later, a significant reduction in stunting prevalence is observed. Some exceptions stand out, however: the district of Netrokona in the Mymensingh region shows a high stunting rate of > = 50% in both periods. The predominant improvement (stunting rate < = 30%) has occurred in the north-west–south parts of Bangladesh e.g. Rangpur, Rajshahi, Khulna, and Barisal with the exception in Gaibandha (> = 50%), and Panchagar (40–50%) from the Rangpur region. A few districts from Rajshahi and Khulna, bordering Dhaka, also made less improvement (stunting rates 30–40%).

**Fig 3:**
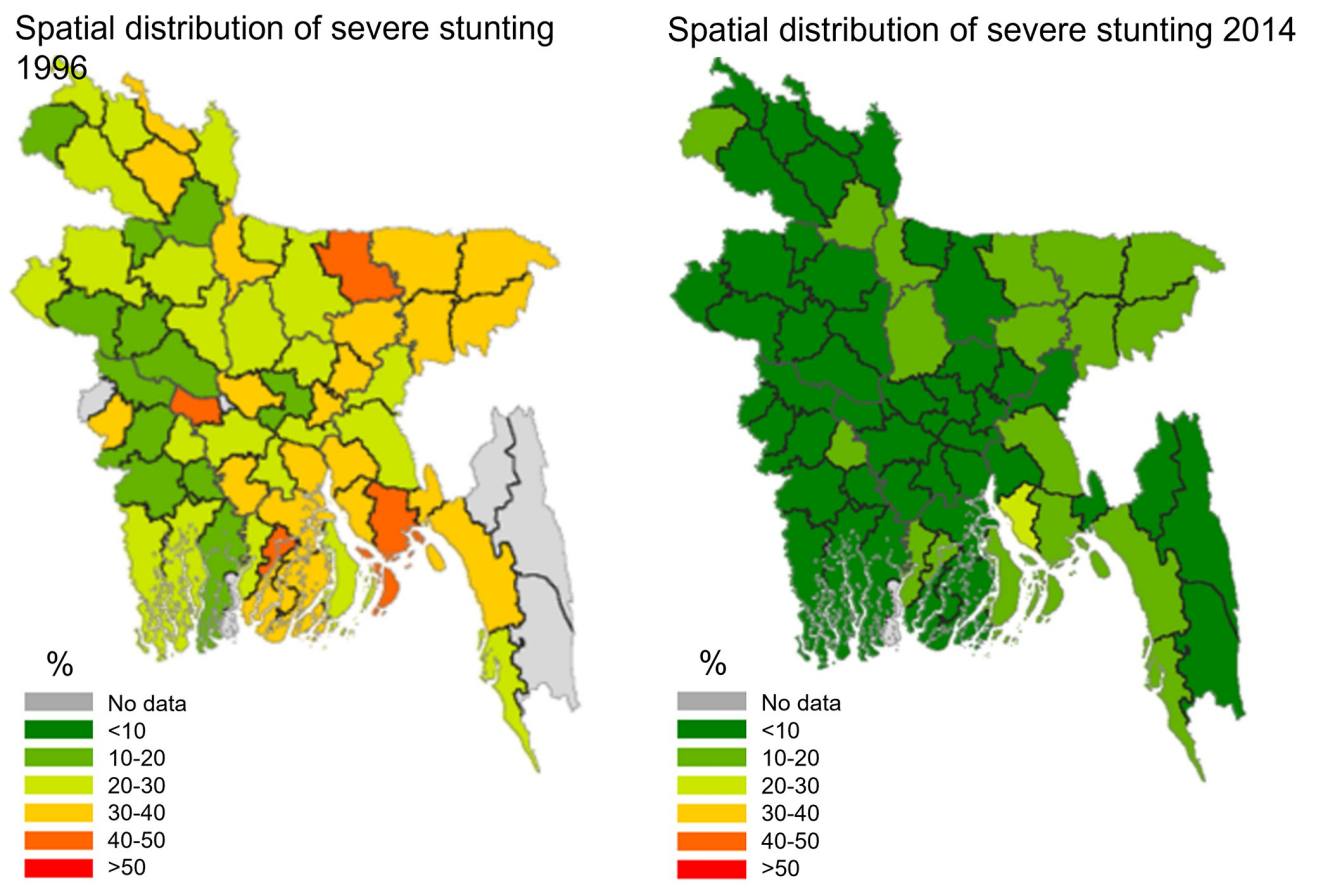
Spatial distribution of severe stunting among under-fives. Source: Own calculations with GRCP software.

**Fig 4:**
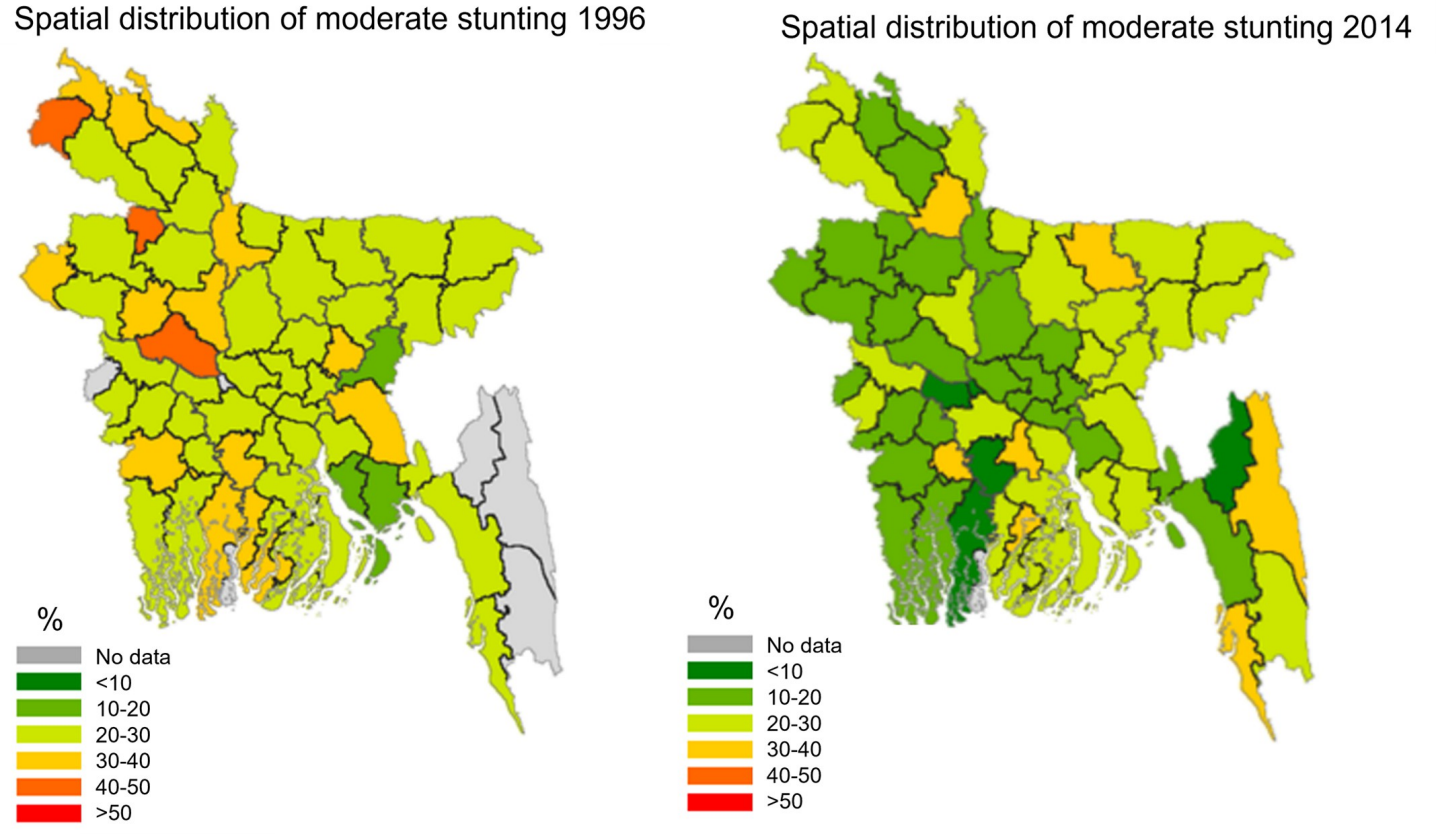
Spatial distribution of moderate stunting among under-fives. Source: Own calculations with GRCP software.

Fig 3 considers severe stunting, while Fig 4 concentrates on moderate stunting. As can be seen in Fig 3, significant improvements can be observed mainly in severe stunting rates. Across Bangladesh the severe stunting rates are below 20%, whereas in 1996/97, such low levels were observed only in some districts of Rajshahi, Rangpur, and Khulna. A massive reduction in severe stunting rates has occurred in the south-east part of Bangladesh. Fig 4 shows that a similar distribution in the changes between 1996/97 and 2014 is observed for moderate stunting rates in Bangladesh. Reductions mainly occurred in Rangpur, Rajshahi, Khulna and Dhaka (areas are next to Khulna). However, there was no change in the moderate stunting rates in Sylhet, and neighboring areas in other divisions e.g., Mymensingh, Barisal, and Comilla.

The distributions of severe, moderate, and total stunting rates throughout Bangladesh and the significant difference between two surveys raise an important question of policy implications and impact evaluation. Which factors play a role in explaining the differences in the reductions of stunting rates among under-fives in Bangladesh? Our analysis explores this to support next steps for policy efforts to achieve the SDG goals in Bangladesh.

### 3.2. Characterizing vulnerable populations

#### 3.2.1. Selection of variables

According to the 1996/97, and 2014 Bangladesh Demographic Health Surveys (BDHS) approximately 60%, 36.2% (WHO new) of children aged 0–59 months old are stunted. Stunting prevalence varies by the sample characteristics, and also by region, and urban-rural residence. In the Annex, the unadjusted covariate effects on the prevalence of stunting and their significant levels in all surveys are shown.

Univariate analysis reveals that children’s residence in urban versus rural areas is significantly associated with stunting probability, although the gap decreases over the years, while gender of children only varies marginally with stunting probabilities in children under-five in all periods. The percentage distribution of stunting varies significantly (p<0.001) by mother’s height, mother’s body mass index (BMI), parent’s schooling years, home delivery versus institutional delivery, and household asset quintiles. The presence of other children under five in the household (indicating lack of family planning) is marginally varying with stunting rates in both periods but is still included in the polling analysis because of the high priority given to family planning in the 4^th^ Health Sector Programme, 2018–2021 (FP2020, 2019) [26]. This leads to the following set of variables qualify for inclusion in the profile: Gender and age of children; education of the mother; education of the father; BMI of mother; height of mother, age of mother at their child’s birth; place of delivery; wealth quintile (rural/urban specific and national); residence (rural/urban), administrative divisions/regions; and number of other children under five in household. We note that S1 Table in the Annex also includes the results for the 2017/18 survey, where in qualitative terms, the same results follow, the only difference being that that the rural/urban gap has decreased. The analysis will address the important key questions why and how the progress made towards reducing the stunting rates over the period of decades; do the covariates play a significant role?

### 3.3. Polling results, 1996 vs 2014

Polling analysis helps to link observed stunting scores to a household *profile*. This implies that all permutations of subsets of five variables from the total set of selected ones are evaluated to assess the likelihood of being associated with the observed stunting scores among under-fives in Bangladesh. Hence, 462 polling analyses are performed, from which winning profiles (profiles with maximum coverage and edge) are selected.

We present our polling results separately for both surveys in Tables 1 and 2.

**Table 1:** Polling results from 1996 (new WHO).

	Profile					coverage	Edge
**Bangladesh**	Rural	mother no education	father no education	child > 1 year	home birth	0.24	2.74
**Barisal**	Rural	Mother primal education	Child male	Child>1 year	Home birth	0.17	1.30
**Chittagong**	Rural	Mother no education	Child>1 year	Home birth	one other children	0.24	2.44
**Dhaka**	Rural	Mother no education	Father no education	Child > 1 year	Home birth	0.26	4.06
**Khulna**	Rural	Mother no education	Father no education	Child> 1 year	One other child	0.19	2.36
**Rajshahi**	Rural	Mother no education	Father no education	Child > 1 year	Home birth	0.33	2.91
**Rangpur**	Rural	Mother no education	Father no education	Child > 1 year	Home birth	0.29	2.58
**Sylhet**	Rural	Mother no education	Father no education	Child > 1 year	Home birth	0.39	3.30
**Mymensingh**	Rural	Mother no education	Father no education	Child> 1 year	Home birth	0.23	2.18

**Table 2:** Polling results from 2014 BDHS (new WHO).

	profile					coverage	Edge
**Bangladesh**	Rural	Normal BMI	Child>1 year	Home birth	one other child	0,14	1,64
**Barisal**	Rural	Male child	Child>1 year	Home birth	one other child	0,19	1,12
**Chittagong**	Rural	Normal BMI	Male child	Child > 1 year	Home birth	0,18	1,46
**Dhaka**	Rural	Normal BMI	Child female	Child > 1 year	one other child	0,16	2,01
**Khulna**	Rural	Normal BMI	Male child	Child > 1 year	one other child	0,20	1,46
**Rajshahi**	Rural	Child female	Child > 1 year	Home birth	one other child	0,18	1,43
**Rangpur**	Rural	Normal BMI	Child male	Child > 1 year	Home birth	0,21	1,85
**Sylhet**	Rural	Poorest	Father no education	Child > 1 year	Home birth	0,16	4,01
**Mymensingh**	Rural	Normal BMI	Father no education	Child > 1 year	Home birth	0,24	3,73

Table 1 shows the winning profiles with coverage (likelihood) and edge (odds ratios) for all (including newly formed Mymensingh division) divisions and for Bangladesh as a whole. In the sample, overall, 24% of all stunted children in Bangladesh are associated with the wining profile of *child is older than one year; residence is in rural area; both parents have no education; and child is born at home*. The likelihood of this wining profile is found to be 2.87 times higher than the likelihood of second-best profile, implying a high confidence level in this association.

However, to target heterogeneous populations for policy making, polling analysis at lower levels of administrative divisions is also included. The national winning profile was observed for five divisions (Dhaka, Mymensingh, Rajshahi, Rangpur, and Sylhet). Chittagong and Khulna deviate from the national wining profile by one variable ‘*more than one under-fives in the HH*’, Barisal deviates by two variables ‘*male child*’ and ‘*mother has primary education*’. The highest coverage (about 39% of all stunted children) is observed in Sylhet, followed by 33% in Rajshahi, 29% in Rangpur and 26% in Dhaka. The odds ratios for all these winning profiles were significantly higher than the likelihood of second-best profiles.

Table 2 reveals the winning profile for children who are scored as stunted in 2014. This profile includes: *child is older than one year; residence is in rural area; maternal BMI is normal; child is born at home; and more than one under-fives in a household*. 14% of all stunted children in the sample are being associated with this profile, and the likelihood of this profile is 1.6 times higher than the likelihood of the second-best profile. This implies that confidence in this association is much lower than that for the winning profile in 1996, pointing at more diversity in the types of households with stunted children than in this earlier period.

The winning profile of being stunted is heterogeneous by different divisions. The winning profile in Mymensingh covers 24% with odds ratio 3.73. The second highest coverage is observed in Rangpur (21%, odds ratio: 1.85). Although the combinations of variables in the profiles were different, *rural residence* and *child older than one year* are uniquely distributed in all winning profiles. *‘Normal BMI’; ‘child is born at home’ and more than one under-fives in a household’* are the second dominating combination of variables in winning profiles. ‘*Poorest household’* and ‘*Father has no education’* were found to be the dominant characteristics of the winning profile that explain childhood stunting only in Sylhet. This diversity in results per region support the national finding that the diversity of households with stunted children has increased, which may be caused by a relatively successful targeting of nutrition policies. Section 4 returns to this point.

### 3.4. Polling results: 2014 vs 2017/18

Finally, we considered the BDHS 2017/18 data on stunting and addressed the question whether this would confirm the dynamics observed in the period 1997–2014. Given the change from targeted to more holistic approaches to decrease stunting, we would expect no significant change in the diversity of the profiles, nor in the profiles themselves. Table 3 presents the polling results for 2017/18.

**Table 3:** Polling results from 2017/18 BDHS (new WHO).

	Profile					coverage	Edge
**National**	Rural	Mother secondary education	Mother normal BMI	Child>1 year	One other child	0,11	1,63
**Barisal**	Rural	Mother normal BMI	Child female	Mother primary education	Child>1 year	0,11	1,36
**Chittagong**	Rural	Mother normal BMI	Child male	Child>1 year	Home birth	0,12	2,10
**Dhaka**	Urban	Mother normal BMI	Child male	Child>1 year	One other child	0,14	1,20
**Khulna**	Rural	Mother secondary education	Mother normal BMI	Child>1 year	One other child	0,16	1,72
**Rajshahi**	Rural	Mother secondary education	Mother normal BMI	Child>1 year	One other child	0,20	3,16
**Rangpur**	Rural	Mother normal BMI	Child male	Child>1 year	One other child	0,15	1,55
**Sylhet**	Rural	Mother normal BMI	Child female	Child>1 year	Home birth	0,12	1,69
**Mymensingh**	Rural	Mother normal BMI	Child>1 year	Home birth	One other child	0,16	2,51

The polling results in Table 3 indeed confirm that the coverage has remained low (and therefore the diversity of profiles high. In fact, for Bangladesh as a whole and for all regions except Rajshahi the coverage fell. Comparing the winning profiles, the results also confirm that by and large, the profiles have remained unchanged.

## 4. Discussion

The country has experienced tremendous change in child nutrition over the past few decades. A significant reduction in childhood stunting was achieved during this period: from a rate of 59% (new WHO) in 1996–97 to 36% (new WHO) in 2014, and 31% in 2017/18 [21]. The Health, Population and Nutrition Sector Development Programme (HPNSDP) of the Government of Bangladesh has set a target to bring down stunting rate at about 25 percent by the period 2016–2021, however, World Health Assembly (WHA) targets this rate to 21.6% by 2025 (NIPORT et al., 2016; Osmani et al, 2016). Using national data BDHS 1996/97,2014, and 2017/18 this study attempted to map such reductions across Bangladesh and to explore the distribution of joint covariates effects (wining profiles) that are associated with childhood stunting over these periods by regions and overall. The results give an insight of the wining profiles covering the period of the first, second and third Health, Population and Nutrition Sector Programs in Bangladesh, as well as covering the start of the implementation of a wide range of nutrition sensitive services and the fourth sector-wide population sector program between 2016–2021.

Overall, the covariates: *‘Parental levels of education’*, *‘children older than one year old’*, *‘child lives in rural area’*, *‘child born at home’* formed the country winning profile in 1996/97, whereas parental levels of education disappear in the winning profile for children who were stunted in 2014. The common variables in the winning profile associated with stunting in both periods are ‘*rural residence’*, *‘child older than one year’*, *and ‘home birth’*. This situation reflects that the country was failing in fulfilling its existing policies see Table 3 of [22] to ensure improved nutrition for all. This finding suggests strengthening the National Policy on Infant and Young Child Feeding, to improve access to nutrition and maternal care services.

The 2014 wining profile includes, instead of “*parental levels of education”*, “*normal maternal BMI”* and ‘*more than one under-fives’* in the HH. This finding provides support for the priority given to strengthening family planning services in the current fourth Health Sector Progamme. In line with common findings in the literature on the detrimental effects of parental schooling years on childhood stunting [13, 14, 17], the likelihood of being stunted was higher among no educated parents in 1997. Interestingly, however, for 2014, winning profiles no longer include parental lack of education. This may point to the fact that over the years, Bangladesh has been successful in addressing parental education for long-term reductions in child undernutrition. The results for 2017/18 confirm the results for 2014, with an increase diversity in profiles and only minor changes in the variables included in the winning profiles, nationally as well as at regional level. Studies evident on the success of women’s education in Bangladesh include e.g. [22, 23]. However, the role of father’s education is underrated, and our findings for 1996 support the conclusion in Vollmer et al (2017) [27] that father’s education is also an important factor in children’s nutritional status. In addition, Saha et al., 2019 [15] specifically stressed the impact of father’s education to bring down the high prevalence of stunting in Sylhet; in our results, it seems that this approach has been successful, as father’s education no longer figures in the dominant profile for 2017 while it did in 1996/1997 and 2014. Generally, the results for 2017 confirm the results for 2014, with an increase diversity in profiles and only minor changes in the variables included in the winning profiles, nationally as well as at regional level.

For the period 1996/97 until 2014, a significant reduction in the distribution of stunting is observed, but this reduction was not uniform across the country. For example, the district Netrokona from the Mymensing region consistently has the highest stunting rates in both periods, and a small reduction was observed in other districts of this region. Further support is evident from our polling results where no changes are observed in the household profiles *“rural residence”*; *“father has no education”*, *“child is older than one year old” “child is born at home”* associated with observed stunting scores between two periods. Besides, geographic location perhaps hinders the well-being of residents in Netrokona which is situated in the northern part of Bangladesh, near the Meghalayan border. This finding calls for necessary action for national nutrition services (NNS) for further reduction of stunting among these vulnerable groups.

The predominant improvement occurred in the eastern-south-western parts of Bangladesh e.g. Rangpur, Rajshahi, Khulna, and Barisal. Diffusion of agrarian and cultural practices between the two Bengali-speaking regions, i.e. West Bengal and Bangladesh, cannot be ignored. We can corroborate this argument with the fact that the bordering districts of southern West Bengal, that are adjacent to Khulna, Rajshahi and a few other districts hold similar demographic and nutritional rates [28]. Needless to mention, there are similarities in the nature of the soil in Khulna, Rajshahi and adjacent districts of West Bengal. These areas are covered with loamy or clay loam soil of the Gangetic plain that has good agricultural productivity, which may explain the relatively better food supply. In addition, there has been sufficient evidence of women’s education, autonomy and decision-making capacity in these regions and thus they excel in child nutrition when compared to the other divisions of Bangladesh. Hence favourable climatic and agricultural environment along with women’s empowerment and knowledge related to child nutrition serve as primary factors to reduce the stunting among children, as compared to northern divisions of the country.

Sylhet remains the worst division of Bangladesh in terms of stunting prevalence. National food security data have placed Sylhet as a relatively food secure region and yet this region has the poorest nutritional status [29]. Although Sylhet is a relatively rich district, poverty coexists with richness [30, 31]. The local geography of Sylhet is also not favourable for year-long agricultural production, due to leached tropical soil, lack of crop rotation and environmental shocks (BBS 2007). Apart from improving economic condition in Sylhet, another key intervention required is improving education levels and economic status of the poor.

A few districts from Rajshahi, Khulna and Rangpur (bordering Dhaka) showed little improvements. This specifically holds for the Gaibandha area of Rangpur. Furthermore, some districts from the southeast part of Bangladesh e.g., Sylhet, Comilla, Mymensingh, Barisal, and Chittagong consistently have the highest percentage of childhood stunting rates. For these regions, there seems to be a clear relation with hunger and poverty reductions, envisaged in the program NFNP of 1997 and other nutrition policies after 1997. It seems that areas similar to Sylhet may have less access to national nutrition services than planned under the Health, Population and Nutrition Sector Development Program and National Nutrition Policy in 2011.

A renewed call for action is needed to address the underlying causes of undernutrition including maternal and paternal education, child marriage and early first birth, sanitation and hand washing practices, access to food and health care, infant and young child feeding practices and the status of girls and women in the family and in society.

In general, the profiles in 2014 are much more diverse than in 1996, which can be explained by the relative success of specific targeted policies in some divisions, while being much less successful in other regions. This would be consistent with the observation that not only stunting levels have not fallen in these areas, but also that the dominant profiles for households with stunted children have remained largely unchanged.

The results for the period 2014–2017 confirm this picture: for Rajshahi, the progress in reducing stunting has been the lowest across Bangladesh (2%), and this is the only region where the coverage of the dominant profile increased, and the edge is also high. For all other regions, the coverage falls, with increases in diversity being strongly correlated with the improvement in stunting. This therefore suggests that the challenge lies in the *implementation* of policies, rather than in the generic approach and focus.

We note that as we had no access to the 2020 DHS data, we cannot account for any impact of Covid-19 on stunting outcomes, although it is, of course, unsure whether the data already capture the effects of the pandemic anyhow. However, given assessments of its impact that are by now available for other countries e.g.,see [32, 33], it is seems likely that there has been a strong negative effect of Covid-19 on stunting, although it is also clear from the literature that targeted interventions can mitigate the negative effects to a large extent. our findings remain valid also for interventions in crisis situations such as the pandemic, as they highlight the importance of access to antenatal and postnatal care, as well as nutritional interventions for children older than 1 year to mitigate the negative impact of these shocks.

## 5. Conclusion

Our study supports the NNP [34], which acknowledges the existing policies of the country related to nutrition but also makes an effort to utilize and incorporate these policies as means to the overall end–an improvement in nutritional status see [19] and Table 2 in [23]. In general, our findings reflect that the access to antenatal and postnatal care, as well as nutritional interventions for children older than 1 year could strongly improve the nutritional status of children. In addition, we also find support for a policy focus that explicitly includes the fathers, particularly when it comes to strengthening education. For specific regions, progress has been significantly slower than average. This in particular holds for Sylhet and areas with similar characteristics, which seemed to have suffered from a lack of access to nutritional services planned under various policy interventions. For such regions, renewed efforts are needed, targeting children older than one year among vulnerable groups, in addition to strengthening family planning programs (because of the association of larger families with stunting prevalence. Regular repetition of the analysis of progress and changes in profiles of households to be targeted would allow not only to track progress, but also to fine-tune policies where needed.

## Supporting information

S1 Table Weighted prevalence of stunting at 0–59 months old children by sample characteristics, 1996/97, 2014, and 2017/18 Bangladesh Demographic Health Survey (BDHS). (DOCX)
