# Supplementary material for: Changes in the determinants and spatial distribution of under-five stunting in Bangladesh: Evidence from Bangladesh Demographic Health Surveys (BDHS) 1996–97, 2014 and 2017/18
Source: PLoS One. 2022 Dec 1;17(12):e0278094. doi: 10.1371/journal.pone.0278094 (PMC9714885; doi:10.1371/journal.pone.0278094)
Supplement: S1 Table — (DOCX) [file pone.0278094.s001.docx]

Table A1: Weighted prevalence of stunting at 0-59 months old children by sample characteristics, 1996/97, 2014, and 2017/18 Bangladesh Demographic Health Survey (BDHS).

| Covariates | BDHS 1996/97 (n=4711) | BDHS 2014 (n=6965) | BDHS 2017/18 (n=7847) |
| --- | --- | --- | --- |
| Stunting | 59.3 | 36.2 | 31.0 |
| Divisions |  |  |  |
| Barisal | 60.1 | 43.1 | 32.8 |
| Chittagong | 54.2 | 38.0 | 32.5 |
| Dhaka | 56.2 | 34.1 | 25.5 |
| Rajshahi | 53.4 | 30.9 | 31.1 |
| Khulna | 47.5 | 27.9 | 25.6 |
| Rangpur | - | 36.6 | 30.2 |
| Sylhet | 61.9 | 50.0 | 42.4 |
| Residence status |  |  |  |
| Urban | 40.2 | 30.8 | 25.3 |
| Rural | 56.1 | 38.1 | 32.7 |
| Gender of child |  |  |  |
| Male | 55.3 | 36.9 | 30.8 |
| Female | 54.4 | 35.6 | 30.7 |
| Age of child (months) |  |  |  |
| 0-6 | 14.5 | 14.1 | 19.8 |
| 6-11 | 31.5 | 19.4 | 20.2 |
| 12-23 | 60.6 | 38.4 | 34.2 |
| 24-35 | 60.2 | 41.2 | 39.0 |
| 36-47 | 65.4 | 45.3 | 33.2 |
| 48-59 | 65.6 | 38.7 | 28.4 |
| Religion |  |  |  |
| Non-Muslim | 51.1 | 35.1 | 28.8 |
| Muslim | 55.2 | 36.3 | 30.9 |
| Institutional delivery |  |  |  |
| Yes | 47.8 | 35.3 | 36.3 |
| No | 57.4 | 37.6 | 28.2 |
| Schooling years of Mother |  |  |  |
| No education | 61.1 | 47.4 | 42.9 |
| Above primary & below secondary | 56.5 | 43.8 | 38.7 |
| Secondary+ | 31.2 | 29.1 | 25.8 |
| Mother’s BMI |  |  |  |
| >18.5 kg/m^2^ | 50.8 | 33.9 | 29.1 |
| <=18.5 kg/m^2^ | 58.6 | 43.6 | 40.1 |
| Mother’s height |  |  |  |
| >145 cm | 52.0 | 33.3 | 27.2 |
| <=145 cm | 68.9 | 56.4 | 54.1 |
| Mother’s age at birth |  |  |  |
| >=20 years | 54.4 | 35.8 | 30.2 |
| <20 years | 55.9 | 37.1 | 31.9 |
| Schooling years of Father |  |  |  |
| No education | 57.8 | 48.1 | 43.6 |
| Above primary & below secondary | 44.0 | 40.6 | 35.8 |
| Secondary+ | 29.1 | 26.3 | 23.3 |
| Household wealth quintals |  |  |  |
| First quintal | 61.5 | 51.3 | 40.7 |
| Second quintals | 62.4 | 40.9 | 37.4 |
| Third quintals | 58.9 | 36.6 | 30.6 |
| Fourth quintals | 53.9 | 31.4 | 26.5 |
| Fifth quintals | 37.1 | 19.6 | 17.4 |
| Under- fives in household |  |  |  |
| 1 | 53.5 | 35.0 | 30.6 |
| >1 | 56.7 | 39.5 | 32.4 |
